# The Efficacy of Nutritional Strategies and Ergogenic Aids on Acute Responses and Chronic Adaptations to Exertional-Heat Exposure: A Narrative Review

**DOI:** 10.3390/nu16223792

**Published:** 2024-11-05

**Authors:** Ryan A. Dunn, Grant M. Tinsley, Ty B. Palmer, Courteney L. Benjamin, Yasuki Sekiguchi

**Affiliations:** 1 Department of Kinesiology and Sport Management, Texas Tech University, Lubbock, TX 79409, USA; dun77277@ttu.edu (R.A.D.); grant.tinsley@ttu.edu (G.M.T.); ty.palmer@ttu.edu (T.B.P.); 2 Department of Kinesiology, Samford University, Birmingham, AL 35229, USA; cbenjami@samford.edu

**Keywords:** exercise, heat acclimation, heat stress, hydration, nutrition, supplement, thermoregulation

## Abstract

Global warming is attributed to an increased frequency of high ambient temperatures and humidity, elevating the prevalence of high-temperature-related illness and death. Evidence over recent decades highlights that tailored nutritional strategies are essential to improve performance and optimise health during acute and chronic exertional-heat exposure. Therefore, the purpose of this review is to discuss the efficacy of various nutritional strategies and ergogenic aids on responses during and following acute and chronic exertional-heat exposure. An outline is provided surrounding the application of various nutritional practices (e.g., carbohydrate loading, fluid replacement strategies) and ergogenic aids (e.g., caffeine, creatine, nitrate, tyrosine) to improve physiological, cognitive, and recovery responses to acute exertional-heat exposure. Additionally, this review will evaluate if the magnitude and time course of chronic heat adaptations can be modified with tailored supplementation practices. This review highlights that there is robust evidence for the use of certain ergogenic aids and nutritional strategies to improve performance and health outcomes during exertional-heat exposure. However, equivocal findings across studies appear dependent on factors such as exercise testing modality, duration, and intensity; outcome measures in relation to the ergogenic aid’s proposed mechanism of action; and sex-specific responses. Collectively, this review provides evidence-based recommendations and highlights areas for future research that have the potential to assist with prescribing specific nutritional strategies and ergogenic aids in populations frequently exercising in the heat. Future research is required to establish dose-, sex-, and exercise-modality-specific responses to various nutritional practices and ergogenic aid use for acute and chronic exertional-heat exposure.

## 1. Introduction

Global warming is associated with an increased frequency of high ambient temperatures and humidity levels [1], elevating incidences of high-temperature-related illness and death [2]. The addition of heightened relative humidity levels to already hot environments causes particular challenges to overall heat storage, as reduced evaporative sweat losses result in a lower magnitude of heat dissipation [1,2]. In combination with elite sport globalisation, higher temperatures worldwide and associated exertional-heat stress compromises exercise performance [3]. This includes aspects such as physical, technical/skill-based, neuromuscular, and cognitive performance [4,5]. Additionally, military training and deployment often involves prolonged, intense activity in environmental extremes, frequently undertaken with insufficient heat preparation [6,7]. Occupational thermal exposure (e.g., firefighting) also reduces work capacity and increases injury risk [8]. Collectively, exercise in hot and/or humid environments exacerbates physiological and thermoregulatory strain, increasing susceptibility to exertional-heat illness/stroke (EHI/EHS) [9]. This can be particularly problematic for cardiovascular function when performing physical activities in the heat, in addition to exacerbating complications arising from various common health conditions (e.g., cardiovascular disease, diabetes). Despite difficulties quantifying the prevalence of EHI/EHS due to inconsistencies in terminology and criteria for diagnosis, endurance sports and military populations experience the highest rates of heat-related illnesses globally, with ~674.0 cases of EHI per 10,000 athlete-exposures [10]. Therefore, exercise performance in the heat can be suboptimal, with tailored heat mitigation and nutritional strategies required to attenuate heat-mediated decrements in performance and health [3].

Heat mitigation strategies such as heat acclimation/acclimatisation (HA/HAs) lessen the magnitude of physiological, thermoregulatory, and perceptual strain associated with exertional-heat stress [11,12]. These include body fluid and electrolyte losses, increased core temperature (T_c_) and heart rate (HR), augmented substrate utilisation, and enhanced thermal discomfort [13,14]. HA/HAs (involving artificial or natural heat exposures, respectfully) deliver regular and controlled disturbances to thermal homeostasis [11], inducing chemical, functional, and genetic adaptations that contribute to attaining a thermotolerant phenotype [12]. Collectively, these adaptations have been shown to benefit exercise performance in the heat by reducing physiological, thermoregulatory, and cellular stress responses during future exertional-heat exposures [9]. 

Although chronic heat adaptation strategies procure the most robust adaptations for optimal performance in the heat, singular heat exposure bouts may also improve some aspects of exercise performance in a timelier manner [15]. Acute heat stress typically hinders exercise performance by augmenting T_c_, HR, and substrate utilisation [16]. Despite this, Ball [17] highlights the positive implications of acute heat exposure, where elevated muscle temperature-related improvements in metabolic and contractile functioning increase adenosine triphosphate (ATP) turnover rates. Although the effects of acute heat exposure on exercise performance likely depend on the modality, duration, and metabolic demand of exercise, a bout of muscle-damaging exercise in the heat was attributed to reduced physiological strain during future exertional-heat stress [18,19]. Additionally, heat shock protein 70/72 expression was diminished prior to and following subsequent exertional-heat stress, indicative of a lower magnitude of heat-induced cellular stress [20]. This highlights the potential for acute heat exposure strategies to assist in the acquisition of a thermotolerant phenotype [18,19,20]. Taken together, tailored acute and chronic heat adaptation methods can potentially enhance exercise performance in the heat. However, it is important to continually develop, evolve, and review these strategies with the aim of maximising adaptation potential [21].

Emerging evidence indicates that nutritional and hydration strategies should complement heat adaptation interventions to enhance overall performance and safety under exertional-heat stress [22]. Therefore, it is important to review the current evidence surrounding these strategies to establish the efficacy and practicality of their use for exercise in the heat [23]. Current reviews, global position stands, and consensus statements regarding nutritional practices to assist with exertional-heat stress have focused on athletes’ knowledge and use of heat adaptation and nutritional interventions, fluid and electrolyte replacement strategies for health and performance, and recovery following heat stress [22,23,24,25]. However, there is a need to evaluate the effectiveness and application of nutritional strategies and ergogenic aids for various exercise settings in the heat. Therefore, the purpose of this narrative review is to (1) discuss how different nutritional practices and ergogenic aids influence responses to acute exertional-heat stress, and (2) discover how adaptations from chronic heat mitigation strategies (e.g., HA/HAs) can be accelerated with the addition of modified nutritional interventions. 

## 2. Search Strategy

All studies investigating the efficacy of various nutritional strategies and ergogenic aids on acute responses and chronic adaptations to exertional-heat exposure were searched through April 2024 in PubMed, SPORTDiscus™, Scopus, and Google Scholar. The keywords used included ‘heat stress’, ‘heat acclimation’, ‘acclimatisation’, ‘thermoregulation’, ‘endurance’, ‘neuromuscular performance’, ‘aerobic performance’, ‘intermittent sports’, ‘team sports’, ‘exercise’, ‘diet*’, ‘ergogenic aid’, ‘supplement’, ‘nutrition’, ‘metabolism’, ‘oxidation’, ‘carbohydrate’, ‘energy availability’, ‘gastrointestinal function’, ‘immune function’, ‘adaptation’, ‘protein’, ‘recovery’, and ‘hydration.’ Subject headings were used independently and in combination. Initial article searching was restricted to full-text articles using human participants, published in English-language, peer-reviewed journals. Randomised control trials, observational, crossover, and interventional studies were included for review. Review articles, abstracts, case studies, unpublished theses, conference papers, and editorials were not included for discussion. The ambient temperature of exercise performance conditions had to exceed 28 °C, simulating predicted environmental temperatures of major sporting events [3]. Additionally, studies incorporating HA/HAs interventions were performed for ≥5 days, representing typical short-term protocols, with a minimum of ≥3 heat exposure sessions conducted per week. Lastly, the induction of HA/HAs-induced adaptations had to be quantified in terms of exposure and duration, with outcome variables measured at least pre- and immediately post-intervention (within 24 h).

## 3. Hydration and Fluid Replacement

### 3.1. Hydration Status and Exertional-Heat Stress

Exertional-heat stress presents numerous challenges for health and performance. In relation to hydration status, exercise in the heat elevates metabolic heat production, whereby increases in sweat rate maintain thermal homeostasis via evaporative fluid losses from the skin [26]. If fluid losses are not sufficiently replaced, reductions in total body water and plasma volume (P_V_) adversely alter cutaneous blood flow, elevate T_c_, and exacerbate cardiovascular responses during exercise [27,28]. Ultimately, this decreases exercise capacity and compromises cardiovascular stability, increasing the susceptibility to EHI/EHS [29]. It is generally accepted that performance impairments occur when fluid losses exceed 2% of body mass [30]. This can be measured by recording the difference between pre- and post-exercise body weight. Additionally, individuals can use urine colour as simple and practical means of evaluating hydration status, with dark coloured urine indicative of potential dehydration. Therefore, specific fluid replacement strategies are essential to commence exercise in a euhydrated state, especially when performed in hot and/or humid environments [31].

### 3.2. Electrolyte Balance and Sodium Loading

Sodium is an essential electrolyte for maintaining fluid and blood volume. However, the majority of commercially available sports beverages contain sodium concentrations that do not match sweat electrolyte losses from exercise, resulting in a negative sodium balance [32]. This was initially demonstrated by Vrijens and Rehrer [33], who showed that the ingestion of a sodium-containing sports beverage decreased plasma sodium and P_V_ relative to pre-exercise following a prolonged, moderate-intensity cycle in the heat (Table 1). The purpose of the lower sodium content in sports beverages is to promote adequate gastric emptying rates, increase beverage palatability, and favour exogenous carbohydrate ingestion, with the aim of delaying glycogen store utilisation [31]. Regardless, most research investigating the efficacy of sodium suggests that it has a direct role in the maintenance and restoration of fluid and electrolyte balance during acute bouts of prolonged, moderate-intensity exercise in the heat, and can minimise associated P_V_ reductions when given the appropriate dose [34,35]. Despite this, the optimal sodium dose to augment performance and health outcomes has yet to be determined.

The magnitude of sodium supplementation required to optimise an acute bout of exercise in the heat is likely dependent on factors such as individual body mass, in addition to exercise modality, duration, and intensity [32]. Hamouti and colleagues [36] identified that water combined with moderate and high levels of sodium (82 and 164 mmol, respectively) enhanced P_V_ by ~5%, compared to ingesting water only, during prolonged exercise in the heat. This expansion in P_V_ allowed for the maintenance of cardiac output and stroke volume during exercise, which in turn, led to a 7.4% improvement in cycling power output during a time-trial in the heat [36]. This highlights the potential of appropriate sodium ingestion for providing cardiovascular stability during exertional-heat stress [36]. 

Ingesting high quantities of sodium prior to an acute bout of exercise in the heat (sodium loading) has been suggested as an effective strategy to optimise performance and thermoregulation [37,38]. To test this hypothesis, previous research assessed the effects of consuming 20–40 mg·kg^−1^ of sodium, 1–2 hrs prior to prolonged exercise in the heat (Table 1 [36,39,40]). This research demonstrated improvements in thermoregulatory responses (lower T_c_, increased sweat rate and P_V_) [36,39], with acute sodium loading found to be equally or more effective than supplementation over consecutive days [37]. Thus, acute sodium loading may be practically beneficial for those with limited opportunities to ingest adequate sodium concentrations prior to and during exercise in the heat. Although the mechanisms underlying sex-specific responses to sodium consumption are not fully elucidated, it should be highlighted that females may better maintain sodium balance compared to males, likely due to heightened sodium sensitivity [38]. Thus, findings related to sodium ingestion for exertional-heat stress in males should in theory extend to female populations, who may potentially exhibit greater benefits due to enhanced sodium sensitivity [38]. Future research should seek to investigate the dose–response relationship of sodium intake, in addition to sex differences, on physiological and cognitive performance measures during exertional-heat stress.

### 3.3. Chronic Heat Adaptation with Permissive Dehydration

Beneficial changes induced by chronic heat adaptation strategies may be influenced by the hydration strategy adopted (i.e., maintained euhydration or dehydration via fluid restriction) [12,41]. Commencing repeated HA sessions in a dehydrated state (fluid loss of ~2–3% of body mass), termed permissive dehydration, may accelerate physiological adaptations due to the interrelationship between heat strain and dehydration [42]. HA undertaken with permissive dehydration can increase plasma vasopressin and aldosterone concentrations, attributed to improved renal water retention and regulation of blood pressure during exertional-heat stress [43,44]. Furthermore, research showed that permissive dehydration during a short-term HA protocol (5 days) in trained athletes increased sweat rate and electrolyte retention, expanded P_V_, and augmented cardiovascular and thermoregulatory adaptations compared to HA sessions with euhydration [45]. These findings have direct implications for HA prescription in exercise settings, with a possible means of accelerating adaptation time course and lessening the time burden associated with chronic heat adaptation protocols [11]. 

Despite potential positive implications of HA with permissive dehydration, athletes and their support teams should be cautious when adopting this strategy, as purposely being dehydrated prior to exercise in the heat can exacerbate EHI/EHS risk. Furthermore, research surrounding HA with permissive dehydration is equivocal, whereby Neal and colleagues [46] concluded that it did not produce superior adaptations compared to sessions conducted with euhydration (Table 1). Likewise, Travers et al. [47] highlighted that HA significantly improved power output during time-trial performance in the heat when sessions were conducted in a euhydrated state (+19 ± 16 Watts; *p* = 0.02), with exercise performance adaptations diminished when HA sessions were performed with permissive dehydration [47]. Additionally, when thermal strain is matched, the time course and magnitude of HA-induced adaptations are largely unaffected by permissive dehydration, compared to maintaining euhydration [47]. From a health perspective, chronic exertional-heat stress with dehydration may be a contributing factor to acute kidney infection and potential chronic kidney disease [47]. The only study to investigate this showed that HA with permissive dehydration offered no further benefit to exercise performance or physiological adaptations; however, no increases in acute kidney infection markers were demonstrated [48]. Collectively, current evidence regarding the effectiveness of commencing HA sessions in a dehydrated state is equivocal and likely to be highly individualised. Additionally, further research is required to assess the true effect of this strategy on markers of kidney function and overall health. 

## 4. Carbohydrates

### 4.1. Substrate Utilisation During Exercise in the Heat

During exertional-heat stress, there is an increased reliance on muscle glycogen and anaerobic metabolism [49], which can deplete endogenous glycogen stores quicker than exercise in temperate climates. As such, carbohydrate intake strategies become increasingly important in line with the elevated magnitude of heat stress and heightened substrate utilisation rates. It should also be highlighted that males are proposed to expend more energy from carbohydrate sources during prolonged exercise in comparison to females (53.1% vs. 45.7% [50]). This is likely due to their higher respiratory exchange ratio and emphasises the importance of appropriate carbohydrate intake prior to and during exercise [50]. Individuals exercising for ≥90 min are recommended to consume 30–90 g·h^−1^ of carbohydrates to maintain blood glucose levels during exercise. Such maintenance may help delay the onset of fatigue and increase exercise capacity in the heat, particularly in male populations [51]. 

### 4.2. Intestinal Permeability and Exogenous Carbohydrate Consumption

Exertional-heat stress also compromises intestinal wall integrity, resulting in detrimental downstream signalling cascades which elicit gastrointestinal disturbances, or in more severe cases, EHI/EHS [52,53]. For example, increases in T_c_ as minimal as 1.3 °C have produced elevations in intestinal permeability and secretions of endotoxins that exacerbate pro-inflammatory immune responses [54]. Exogenous carbohydrates may protect against intestinal permeability, where increased production of short-chain fatty acids induce the formation of plaque and membrane proteins that enhance epithelial barrier integrity [54]. In relation, Snipe and colleagues [55] highlighted that ad libitum carbohydrate ingestion during 120 min of submaximal exercise in the heat (60% maximal oxygen uptake [VO_2max_]) ameliorated intestinal epithelial injury, small intestine permeability, and enhanced anti-endotoxin antibody responses when compared to water ingestion alone. Additionally, 30–90 g·h^−1^ of carbohydrates can attenuate pro-inflammatory cytokine production during exercise, indicative of reduced oxidative stress and inflammatory markers [56]. Taken together, it appears that carbohydrate intake during exercise in the heat is important for lowering glycogen depletion rates, and has direct implications for reducing the susceptibility of EHI/EHS, primarily via intestinal protective mechanisms [54].

With most nutritional strategies, the appropriate method and dosage of supplementation is essential to provide the desired effect. Of note, Sessions and colleagues [57] demonstrated that 26 g of carbohydrate ingestion during exercise in the heat enhanced markers of gastrointestinal wall damage, which may exacerbate EHI/EHS symptoms (Table 1). However, carbohydrates were administered via gels, which may indicate that more concentrated ingestion methods alter gastrointestinal function to a higher extent than diluted carbohydrate sources (e.g., carbohydrate containing beverages) [57]. Ultimately, carbohydrate supplementation should be closely monitored during training/practice sessions in the heat to assess the efficacy of their use on markers of gastrointestinal distress and performance [51]. 

### 4.3. Carbohydrate Loading for Exercise in the Heat

Carbohydrate-loading strategies are often implemented to maximise muscle and liver glycogen availability prior to prolonged endurance exercise. Individuals are recommended to consume 10–12 g·kg^−1^ of carbohydrates, 36–48 h before competition [58,59]. During exercise in the heat, redistribution of blood flow to the skin from vital organs and elevations in muscle temperature promoting glycogenolysis likely reduce exogenous carbohydrate utilisation rates [60]. This highlights the importance of adequate carbohydrate intake prior to exercise in the heat. It is yet to be determined if HA/HAs play a role in ameliorating heat-mediated reductions in exogenous carbohydrate oxidation rates [60]. However, combining glucose- and fructose-based carbohydrates (i.e., multiple transportable carbohydrates) promotes higher exogenous carbohydrate oxidation rates than glucose ingestion alone [61]. Therefore, individuals are recommended to ingest combined carbohydrate sources to maximise oxidation rates and extend exercise capacity in the heat [61].

### 4.4. Carbohydrate Periodisation and Exertional-Heat Stress

In contrast to carbohydrate loading, periodisation strategies, such as training with low carbohydrate availability, are employed to increase the efficiency of utilising available fuel sources during exercise [62,63]. Training with low carbohydrate availability potentially enhances fat oxidation rates and skeletal muscle signalling, which have been attributed to improvements in endurance running in both sexes [64]. As exercise in the heat increases carbohydrate metabolism rates, the combined effect of exertional-heat stress and low carbohydrate availability may alleviate reductions in carbohydrate oxidation rates [65]. As such, Bennett and colleagues [66] investigated the potential role of exertional-heat stress and low carbohydrate availability on optimising substrate metabolism during exercise. The authors identified that under exertional-heat stress, endurance training completed with low carbohydrate availability did not improve performance in hot or temperate conditions and failed to increase fat oxidation rates in males [66]. It is yet to be determined if these findings will also extend to females, who are suggested to have higher relative fat oxidation rates than males during exercise [54]. Although exercising with low carbohydrate availability has exhibited positive benefits in temperate conditions, it appears that this strategy requires further study to provide a rationale for its use during exercise in the heat [54]. 

## 5. Protein

### 5.1. Protein and Carbohydrate Supplementation and Exercise Performance

Despite not being directly related to one another in terms of their function within the body, there is growing interest surrounding the additive use of protein to carbohydrates (CHO + PRO) to further augment exercise performance [67]. Initial research assessed whether combined carbohydrate and protein supplementation (CHO, 3.6 kcals·kg^−1^; PRO, 0.36 g·kg^−1^) during a 5-day aerobic training protocol in a thermoneutral environment could enhance physiological and thermoregulatory adaptations [68]. The authors identified that consuming CHO + PRO expanded P_V_ two-fold higher and reduced T_c_, which was associated with reduced thermoregulatory and cardiovascular strain during future exertional-heat stress [68]. Increases in P_V_ were attributed to elevated plasma albumin content, subsequently enhancing plasma oncotic pressure to move fluid from the extra- to intravascular space [68]. These findings support the potential for integrating CHO + PRO supplementation during HA/HAs sessions to enhance cardiovascular and thermoregulatory adaptations.

Combined CHO + PRO supplementation may also be an effective nutritional strategy for exertional-heat stress. Cathcart and colleagues [67] demonstrated that CHO + PRO ingestion (PRO, 18 g·L^−1^; CHO, 76 g·L^−1^) helped maintain individual body mass during a prolonged, multi-day cycling competition in the heat compared to consuming carbohydrates alone. The combination of carbohydrates and protein may mitigate the negative effects of dehydration and associated body mass loss [67]. Additionally, Cathcart et al. [67] found an attenuated rise in tympanic temperature, with athletes completing the race 12% more quickly than those supplementing with only carbohydrates (Table 1 [67]). This was the first study to assess the ergogenic benefits of CHO + PRO ingestion on prolonged exercise in the heat [67]. However, this study’s mitigation of body mass loss may, in part, be due to an increased caloric delivery during exercise resulting from additional protein, with a potential protein-mediated expansion of body water compartments [69]. 

Currently, studies reporting an ergogenic effect with CHO + PRO supplementation during exercise in the heat have typically involved conditions where carbohydrate delivery was suboptimal [70]. When carbohydrate delivery is ≥60 g·h^−1^, the addition of protein has shown no ergogenic advantage during time-to-exhaustion [71] or time-trial performance tests [72]. Consequently, a lack of robust evidence exists to support the additive use of protein to exogenous carbohydrates for enhancing endurance exercise performance when carbohydrate delivery is optimal [73,74,75]. Additionally, most studies reporting exercise performance improvements have used beverages matched for carbohydrate content, but not total energy content, making it unclear whether benefits stemmed from protein-stimulated mechanisms or from extra energy content within the CHO + PRO beverage [76]. Additionally, there is a lack of consideration of the potential influence of female hormonal fluctuations on muscle protein synthesis, whereby changes in ovarian hormones have the potential to inhibit muscle protein synthesis [67]. This is a particular concern for the maintenance of body mass and muscle recovery throughout exertional-heat stress [67]. Taken together, differing methodologies across studies, including experimental design, exercise performance measures, alterations in hormone secretion, and whether control groups were matched for caloric or carbohydrate content, are likely to account for the equivocal findings on CHO + PRO supplementation across the literature. 

### 5.2. Protein and Carbohydrate Supplementation for Recovery Following Exertional-Heat Stress

Although CHO + PRO ingestion warrants further investigation to fully elucidate its role in eliciting physiological and performance adaptations during acute and chronic heat exposure, this strategy may exert the most beneficial effect on exertional-heat stress recovery [77]. Several studies demonstrated either increased muscle glycogen restoration mediated by augmented insulin concentration [77,78,79] or reduced markers of muscle damage [74,80,81,82,83] when CHO + PRO is ingested during or following exercise in the heat compared to CHO alone. The co-ingestion of CHO + PRO likely exerts positive influences on net protein balance via increased protein synthesis and reduced protein degradation, which may be particularly important for females experiencing reductions in muscle protein synthesis due to ovarian hormone fluctuations during their menstrual cycle [84]. Furthermore, exogenous carbohydrates stimulate the release of insulin, which has previously been shown to enhance muscle protein synthesis and muscle fibre repair [81]. Hall and colleagues [85] identified that CHO + PRO consumption (PRO, 0.23 g·kg^−1^·hr^−1^; CHO, 0.87 g·kg^−1^·hr^−1^) during a 2.5 h, high-intensity training session elicited a 1.8% improvement in subsequent cycling time-trial performance in the heat (compared to carbohydrates alone) following a 4 h recovery period. CHO + PRO ingestion was also associated with significant reductions in HR, perceived exertion, and markers of muscle damage, indicating a potential role in reducing physiological and perceptual stress measures during exertional-heat stress, in addition to accelerating muscle recovery [85].

Due to conflicting results, current evidence suggesting that CHO + PRO ingestion can enhance exercise performance is unclear. Despite this, as exertional-heat stress exacerbates carbohydrate utilisation, combining carbohydrates with protein may be more beneficial for exercise in the heat, as optimal carbohydrate oxidation rates are likely more difficult to achieve [85]. Additionally, CHO + PRO ingestion has been shown to improve exercise recovery in both sexes without negatively influencing performance. Therefore, CHO + PRO may be a beneficial strategy for recovery during and following exertional-heat stress. In accordance with established guidelines, male and female athletes are recommended to consume 1.4–2.0 g·kg^−1^ of protein daily, with an optimal protein dose of 20–40 g every 3–4 h to facilitate muscle protein synthesis and optimise recovery [86]. 

**Table 1:** Summary of studies investigating the effect of nutritional strategies on acute responses to exertional-heat exposure and chronic adaptations from heat acclimation.

Study	Participant Characteristics	Supplementation Protocol	Performance Testing	Outcome
*Sodium*				
Vrijens & Rehrer [33]	Endurance-trained males (*n* = 10; age, 25 ± 3 years)	Na^+^-containing (18 mmol·L^−1^) sports beverage	3 hr cycle at 55% VO_2max_ (34 °C; RH, 65%)	↓ Plasma Na^+^ relative to pre-exercise ↓ P_V_ (−9.7 ± 6.6%)
Hamouti et al. [36]	Trained male cyclists (*n* = 10; age, 33 ± 6 years)	High Na^+^ (164 mmol), moderate Na^+^ beverage (82 mmol), or water only	1 hr cycle at 63% VO_2max_ (33 °C; RH, 30%) preceding cycling TT	↓ P_V_ loss with high (−9.8 ± 4.2%) and moderate Na^+^ (−11.9 ± 2.4%) vs. water alone (−16.4 ± 3.2%) ↑ 7.4% in cycling power output vs. water alone
Sims et al. [39]	Trained female cyclists (*n* = 13; age, 26 ± 6 years)	High Na^+^ (164 mmol) or low Na^+^ (10 mmol) beverage	Cycling to exhaustion at 70% VO_2peak_ (32 °C; RH, 50%)	↑ TTE in high (98.8 ± 25.6 min) vs. low Na^+^ (78.7 ± 24.6 min) ↓ Rise T_c_ in high (1.2 ± 0.2 °C) vs. low Na^+^ (1.6 ± 0.2 °C)
Sims et al. [40]	Endurance-trained male runners (*n* = 8; age, 36 ± 11 years)	High (164 mmol) or low Na^+^ (10 mmol) beverage	Running to exhaustion at 70% VO_2max_ (32 °C; RH, 50%)	↑ TTE in high (96.1 ± 25.6 min) vs. low Na^+^ (78.7 ± 24.6 min) ↓ Mean T_c_ in high (38.9 °C) vs. low Na^+^ (39.3 °C) ↑ P_V_ in high (+4.5 ± 3.7%) vs. low Na^+^ (+0.0 ± 0.5%)
***Permissive Dehydration***				
Neal et al. [46]	Trained male athletes (*n* = 8; age, 21 ± 3 years)	5 days of HA with or without fluid restriction	Exertional-heat stress tests (40 °C; RH, 50%)	- No further benefit of HA with fluid restriction on adaptation induction and decay
Garrett et al. [45]	Aerobically fit males (*n* = 9)	5 days of HA with or without fluid restriction	90 min cycling (35 °C; RH, 60%)	↑ PV following HA with DEH vs. EUH
Travers et al. [47]	Healthy males (*n* = 7; age, 34 ± 4 years)	10 days of HA with or without fluid restriction	90 min running (40 °C; RH, 40%)	- No difference between HA with DEH vs. EUH on haematological or cardiovascular measures
Haroutounain et al. [48]	Moderately-trained males (*n* = 14; age, 25 ± 0.5 years)	7 days of HA with or without fluid restriction	16 km running TT (22 °C; RH, 40%)	- No difference between HA with DEH vs. EUH on TT performance or acute kidney infection markers
***Carbohydrates***				
Sessions et al. [57]	Active subjects (*n* = 7)	27 g of CHO gel or non-CHO placebo	2 × 60 min running at 70% VO_2max_ (30 °C)	↑ Endotoxins, TNF-α, and IL-6 post-exercise in CHO gel trial vs. non-CHO trial
Bennett et al. [66]	Endurance-trained males (*n* = 23; age, 30 ± 6 years)	14 days of HA with low CHO intake	30 min exertional-heat stress test (35 °C; RH, 50%)	↓ Fat oxidation rates vs. low CHO training without heat stress
Snipe et al. [55]	Endurance runners (*m* = 6, *f* = 5)	Water or 15 g of CHO	120 min at 60% VO_2max_ (35 °C)	↓ Intestinal injury and permeability markers with CHO ingestion vs. water only ↑ Anti-endotoxin markers with CHO ingestion vs. water only
***Carbohydrates + Protein***				
Goto et al. [68]	Healthy males (*n* = 18; age, 23 ± 4 years)	5 days of aerobic training with or without CHO + PRO	30 min cycling daily at 70% VO_2peak_ (30 °C; RH, 50%)	Greater P_V_, stroke volume, and sweat rate increase in CHO + PRO group vs. control
Cathcart et al. [67]	Trained mountain bikers (*m* = 24, *f* = 4; age, 32 ± 1 years)	CHO (72 g·L^−1^) + PRO (18 g·L^−1^) or matched-CHO PLA (76 g·L^−1^)	8 day, multi-stage mountain bike race (33 °C; RH, 42%)	- No body mass change in CHO + PRO condition ↓ Rise in tympanic temperature in CHO + PRO vs. PLA ↓ Performance time in CHO + PRO (2277 ± 127 min) vs. PLA (2592 ± 68 min)

Na^+^, Sodium; VO_2max_, Maximal oxygen uptake; RH, Relative humidity; P_V_, Plasma volume; TT, Time trial; VO_2peak_, Peak oxygen uptake, TTE, Time-to-exhaustion; T_C_, Core temperature; HA, Heat acclimation; W, Watts; DEH, Dehydration; EUH, Euhydration; CHO, Carbohydrate; PRO, Protein; TNF-α, Tumour necrosis factor alpha; IL-6, Interleukin 6.

## 6. Caffeine

### 6.1. Mechanism of Action

During exercise, increased physiological strain causes disturbances to normal central nervous system (CNS) function [87], likely resulting from an imbalance in neurotransmitters. These effects are increasingly worsened with elevations in ambient temperature and/or humidity [88]. To counteract the deleterious effects of exertional-heat stress on the CNS, caffeine (1,3,7-trimethylxanthine) supplementation may be beneficial [89]. The application of caffeine on exercise performance in temperate conditions has been extensively studied; however, data regarding caffeine’s ergogenic effect on exercise performance in the heat are limited and inconsistent [90]. Although the precise mechanism by which caffeine acts to enhance exercise performance is not fully elucidated, evidence suggests that a blockage of adenosine receptors in the CNS delays the onset of central fatigue during exercise via reductions in the inhibitory effects of adenosine on neuroexcitability and neurotransmitter release [91]. An increase in adenosine binding to its receptors acts as a CNS depressant, leading individuals to feel heightened sensations of sleepiness and reduced arousal [91]. Therefore, caffeine consumption has the potential to increase motivation, drive, and alertness, in addition to lowering perceived exertion during exercise, and enhancing the mobilisation of intracellular calcium and free fatty acids [92]. Collectively, caffeine ingestion may positively influence neuromuscular and cognitive performance measures via reductions in central fatigue development (failure to sustain CNS drive to working muscle), subsequently improving exercise tolerance in the heat. 

### 6.2. Caffeine Supplementation for Exercise Performance in the Heat

The meta-analysis of Naulleau and colleagues [92] concluded that consuming 6 mg·kg^−1^ of caffeine, ~1 h prior to exertional-heat stress may improve exercise performance; however, performance changes were deemed insignificant. Furthermore, the majority of included studies showed modest increases in T_c_ during exercise following caffeine supplementation compared to placebo groups [93]. Initial field studies in this area demonstrated that caffeine had no benefits on endurance exercise performance when tested in hot environments (Table 2 [94,95]). Similarly, the first laboratory-based study to assess caffeine’s effect on acute exertional-heat stress identified no improvement in endurance exercise performance [87]. Studies showing no ergogenic effect of caffeine on exercise performance in the heat (Table 2 [87,96,97]) have attributed the lack of findings to the potential role of caffeine on neurotransmitters, where an increase in noradrenaline secretion may diminish ergogenic effects [97]. Additionally, Hanson and colleagues [96] demonstrated increased rates of heat storage following caffeine ingestion, even in the absence of performance improvements. This has direct implications for EHI/EHS susceptibility; however, further research using dopamine reuptake inhibitors is required to fully elucidate the effects of varying caffeine dosages on neurotransmitter bioavailability and subsequent thermoregulatory responses [96]. 

The majority of studies showing no ergogenic effect of caffeine for exercise in the heat have only examined cycling-based performance measures [87,96,97]). As caffeine likely improves endurance exercise through CNS-related mechanisms, factors such as central fatigue are considered more critical performance-limiting factors in endurance running compared to cycling [98]. Therefore, running test protocols are likely more appropriate for detecting the potential endurance-enhancing effect of caffeine in hot environments. In relation, Ping et al. [99] demonstrated improvements in endurance running performance in the heat following 5 mg·kg^−1^ of caffeine administration (Table 2). Although performance benefits were observed (31.6% increase in time-to-exhaustion), the subjects were heat acclimated individuals; thus, the benefits of caffeine on exertional-heat stress may only be observed when individuals previously undergo chronic heat adaptation [99]. Overall, it is difficult to truly assess the effect of caffeine on exertional-heat stress due to conflicting results across studies investigating differing performance measures, supplementation doses, and participants with varying acclimation/acclimatisation status [93]. Additionally, there are prominent interindividual differences in caffeine metabolism at the genetic level [100]. Individuals with homozygous A/A alleles are categorised as rapid caffeine metabolisers, whereas C allele carriers (A/C and C/C) are known to be slow metabolisers of caffeine [100]. As genetic polymorphisms regarding caffeine metabolism are often overlooked in studies, it is difficult to propose robust conclusions on the effect of acute caffeine ingestion on exertional-heat stress, in addition to providing suggested doses. This is due to the differing rates of caffeine metabolism between participants, with acute prescription of the ergogenic aid typically administered ~1–2 h prior to exercise performance. Despite this, the major consensus regarding caffeine supplementation is that it does not lead to performance decrements in the heat; however, observed increases in T_c_, HR, and blood lactate warrant careful consideration on its use in competitive settings. 

## 7. Nitrate

### 7.1. Mechanism of Action

Another ergogenic aid that may improve exercise in the heat is nitrate (NO_3_^−^) [101]. NO_3_^−^ is a signalling molecule required for the synthesis of nitric oxide (NO), a primary regulator of arterial pressure [102]. Additionally, NO can be catalysed via redox reactions by NO synthase, which can also degrade L-arginine to produce NO. These downstream signalling cascades facilitate the vasodilation of blood vessels and increase blood flow to the working musculature, subsequently lowering blood pressure [103]. NO_3_^−^ supplementation is also thought to improve mitochondrial coupling efficiency, which may increase ATP resynthesis and enhance exercise capacity [104]. Although the mechanisms are not fully elucidated, nitrate also has the potential to improve aspects of cognitive function via increased perfusion to the brain [105]. 

### 7.2. Nitrate Supplementation for Exercise in the Heat

As mentioned, most studies examining ergogenic effects on performance and health are primarily conducted in temperate environments [106,107]. In such environments, pre-exercise consumption of NO_3_^−^ (300–1050 mg, 2–3 h before exercise [108]) has improved performance during sub- and supra-maximal intensity endurance exercise [106,107,109,110], and repeated sprint running [111]. This has been attributed to reductions in ATP cost during muscular contractions and reduced oxygen uptake requirements for mitochondrial ATP resynthesis [112]. Furthermore, NO_3_^−^ has increased vascular conductance and reduced blood pressure following beetroot supplementation (~9.2 mmol·L^−1^) in response to locally administered thermal skin stimuli [102]. Therefore, it is surprising that previous studies investigating the effects of dietary NO_3_^−^ on exertional-heat stress have shown no improvements in performance or thermoregulatory responses (Table 2 [113,114,115,116,117]). However, it is important to note that the source and relative quantity of nitrates within beetroot supplements are highly variable, which can cause discrepancies in findings amongst studies [114]. Initial research demonstrated that 6 days of nitrate-rich beetroot juice (8.4 mmol·L^−1^) reduced oxygen consumption and increased T_c_ during the latter stages of a 45 min march in the heat [113]. Likewise, McQuillan and colleagues [116] identified that short-term NO_3_^−^ supplementation (8.0 mmol·L^−1^ for 3 days) increased T_c_ throughout low- to moderate-intensity cycling exercise in the heat, in addition to offering no further benefit to time-trial performance. As blood flow distribution is altered under heat stress to maintain thermal homeostasis, increases in T_c_ may be a direct consequence of NO_3_^−^ supplementation. NO_3_^−^ is suggested to alter blood flow by enabling whole-body vasodilation and overriding the homeostatic response to exertional-heat stress [110]. Ultimately, NO_3_^−^ may increase the rates of hyperthermia development and hinder thermoregulatory mechanisms.

The absence of a positive change in performance from nitrate supplementation throughout studies may be due to inconsistencies in adopted research designs, sub-optimal conditions for evaporative cooling, supplementation method, or incomplete analyses of thermoregulatory processes [102]. For example, the NO pathway is potentially initiated more efficiently at higher exercise intensities; therefore, the lower continuous exercise intensities utilised in most studies (45–60% VO_2max_) could have limited the actions of the NO_3_^−^ pathway on adequate heat transfer [113,114,117]. Furthermore, as training status can account for variability in performance responses to NO_3_^−^ supplementation [118], Fowler and colleagues [102] investigated the effects of dietary NO_3_^−^ supplementation in untrained males. In line with previous findings, 5 days of NO_3_^−^ supplementation (9.2 mmol·L^−1^) had no beneficial effect on exercise tolerance and thermoregulation in hot, dry conditions [102]. Although the consumption of NO_3_^−^ for exercise performance in temperate environmental conditions seems warranted, there is a lack of robust evidence to support its use for exercise in the heat. It would be beneficial for future studies to explore the efficacy of nitrate supplementation for exercise performance in the heat in trained vs. untrained individuals, in addition to comparing between different sport disciplines (e.g., intermittent and endurance sports). Lastly, current findings have potential implications for HA/HAs protocols, where the ingestion of NO_3_^−^ during HA/HAs sessions may allow individuals to attain the desired thermal stimulus at a faster rate, consequently reducing the time burden associated with chronic heat adaptation. 

## 8. Tyrosine

### 8.1. Effects of Heat Stress on Brain Neurotransmitters

Exertional-heat stress alters the activity and synthesis of the central monoamines, serotonin (5-HT), dopamine (DA), and noradrenaline [119]. An elevated ratio of brain DA to 5-HT may augment performance, whereas low ratios induce lethargy and motivation deficits [120]. This likely results from the well-established role of DA in motor initiation and control, and increased motivation and arousal [121]. In relation to this, previous research demonstrated that increasing brain DA availability improved exercise performance in hot (30 °C), but not temperate (18 °C) conditions [122], suggesting a specific role for DA in subjective exercise tolerance under heat stress. Enhancing DA availability appears to dampen inhibitory signals within the CNS, allowing both males and females to tolerate a higher degree of physiological and thermoregulatory strain at the same perceived exertion [122]. Taken together, increasing brain DA availability may provide a neurobiological mechanism for fatigue development during exercise in the heat, with substances that increase DA availability hypothetically improving exercise capacity in the heat in both sexes.

### 8.2. The Role of Tyrosine for Increasing Dopamine Availability

Administration of the amino acid tyrosine, a dopamine precursor, increases the ratio of tyrosine to other large neutral amino acids (LNAA; leucine, isoleucine, valine, methionine, threonine, lysine, tryptophan) [123]. As tyrosine shares a common transport molecule with LNAA at the blood–brain barrier, elevating the tyrosine ratio increases brain tyrosine and decreases LNAA. Large quantities of LNAA can reduce the uptake of tyrosine into the brain, which has direct consequences on DA and catecholamine synthesis, as tyrosine acts as both a DA and catecholamine precursor [124]. Acute tyrosine supplementation potentially improves measures of mood, DA-dependent cognition, psychomotor performance, and behaviour [124]. As exercise in the heat represents a specific demand on brain DA, which is not apparent in temperate conditions [122,125], the requirement for brain tyrosine may be greater with the cumulative demands of exercise under heat stress, with the potential to limit DA synthesis and release. 

### 8.3. Tyrosine Supplementation for Exertional-Heat Stress

Initial research investigating the effects of tyrosine supplementation for exercise in the heat demonstrated that acute doses of 150 mg·kg^−1^, ~1 h pre-exercise, were associated with increased exercise capacity (~15%) during constant-load cycling in the heat (Table 2 [121]). This novel finding supported the hypothesis that increasing tyrosine availability contributes to improvements in exercise capacity in the heat, and indirectly supports the importance of brain DA in exercise tolerance under heat stress [122,125]. The authors attributed these positive findings to underlying mechanisms of central fatigue in the heat, which can reduce states of arousal [126], concomitant with a linear increase in perceived exertion [14]. A high brain DA:5-HT ratio may preserve arousal levels during prolonged exercise, with increased brain DA availability likely involved in increasing exercise tolerance in the heat [122,125]. These mechanisms may be linked to the observed performance improvements following acute tyrosine supplementation [121]. 

The initial findings of Tumilty and colleagues [121] prompted further research to investigate the role of tyrosine for exercise performance in the heat. In contrast, further studies highlighted that 150 mg·kg^−1^ of tyrosine did not enhance cognitive function or physical performance in hot environmental conditions (Table 2 [127,128,129]). The lack of observed effects may relate to the potential for tyrosine to increase central noradrenaline activity, consequently counteracting any influence on performance [128]. This theory is supported by Roelands and colleagues [130], who emphasised that noradrenaline reuptake inhibitors reduced exercise capacity in the heat. As mentioned, tyrosine acts as a catecholamine precursor, whereby an increase in plasma tyrosine levels potentially stimulates the production of noradrenaline [124]. Taken together, the optimal dose to maximise plasma tyrosine concentration is likely 150 mg·kg^−1^; however, there is a lack of sound evidence to inform its use for improving aspects of exercise performance in the heat. 

## 9. Creatine

### 9.1. Creatine Storage, Synthesis and Dosage

Creatine (methylguanidine acetic acid) is a nitrogenous compound synthesised in the body and remains a widely used ergogenic aid by athletes and recreational exercisers [131]. The majority of creatine resides within skeletal muscle (~95%) as phosphocreatine (PCr), which undergoes rapid dephosphorylation to resynthesise ATP, with small amounts present in the brain (~5%) [132]. Approximately two-thirds of intramuscular creatine is contained as PCr, with the remaining being free within creatine pools [132]. Additionally, ~1–2% of intramuscular creatine per day is degraded into the metabolic byproduct creatinine and excreted through urine [131]. Therefore, the body needs to replenish ~1–3 g of creatine daily to allow creatine stores to be maintained at a homeostatic level [133]. Evidence suggests that ingesting 5 g of creatine monohydrate (~0.3 g·kg^−1^) four times daily for 5–7 days is the most effective way to increase intramuscular creatine stores [134]. However, smaller doses (2–5 g or 0.3 g·kg^−1^) are also effective at maintaining intramuscular creatine stores [135]. Therefore, it is accepted that creatine loading may not be necessary, although this approach remains the most rapid means of increasing intramuscular PCr levels [136]. 

### 9.2. Mechanism of Action

In addition to its consumer popularity, the potential for creatine to enhance exercise performance has been widely researched in sport nutrition literature over recent decades [137]. Creatine supplementation is commonly reported to enhance short-duration, high-intensity activities and promote increased gains in strength, muscle mass, bone mineral density, and neuromuscular function [136]. During short-duration, high-intensity activities, energy for ATP resynthesis is supplied from the breakdown of PCr and anaerobic glycolysis [138]. In addition to increasing concentrations of circulating free creatine and maximising PCr stores within skeletal muscle cells [138], creatine supplementation can also enhance the efficiency of ATP utilisation, subsequently increasing anaerobic power production [138].

Increases in total and lean body mass are commonly reported following short-term creatine supplementation [139]. As creatine is osmotically active, increased concentrations within skeletal muscle elevate intracellular water volume, with short-term fluid retention attributed to acute increases in body mass [140]. Increases in intracellular osmotic pressure promote water movement into the cell, thereby increasing cellular volume [141]. Increases in intracellular volume, likely matched with reductions in extracellular volume, may influence thermoregulatory mechanisms during sustained exercise in the heat [141]. Consequently, the proposed mechanisms of action for creatine prompted interest in determining if supplementation could improve exercise in the heat, likely due to increased fluid retention [142]. 

### 9.3. Creatine Supplementation for Exercise Performance in the Heat

The ergogenic effects of creatine for exercise performance in the heat appear to be related to creatine-induced increases in intracellular water [143]. These increases may help optimise hydration status, improve thermoregulatory responses, and enhance overall heat storage capacity [144]. Concerns with the application of creatine for exercise in the heat were initially raised due to potential P_V_ reductions via decreases in extracellular water, exacerbating cardiovascular responses during exercise [145]. However, the meta-analysis of Lopez and colleagues [132] identified that none of the included studies demonstrated adverse creatine-induced changes in thermoregulatory mechanisms, percentage of dehydration, urinary hydration measures, or P_V_. The lack of influence on thermoregulatory processes was still found despite 90% of included studies reporting an increase in body mass following creatine supplementation [132]. 

Initial research identified that 20 g·day^−1^ of creatine for 7 days prior to a time-to-exhaustion in the heat trial increased intracellular water volume and improved thermoregulatory and cardiovascular responses to prolonged exercise [144]. Creatine supplementation was suggested to promote hyperhydration amongst participants, subsequently eliciting more efficient thermoregulatory processes during prolonged exercise in the heat and limited dehydrated-mediated decrements in performance. Similarly, Volek and colleagues [142] identified that 0.3 g·kg^−1^ of creatine augmented repeated sprint cycling performance in the heat without exacerbating thermoregulatory responses. These results were also replicated by other research assessing the influence of creatine supplementation for exercise in the heat (Table 2 [143,146,147]). Interestingly, similar findings were exhibited in trained, dehydrated men following short-term creatine supplementation (21.6 g·day^−1^ for 7 days), whereby hydration status and thermoregulatory processes were not compromised [145]. Collectively, these findings provide strong evidence that creatine supplementation may be an effective nutritional strategy for athletes engaged in high-intensity exercise in hot and humid environments, without hindering thermoregulatory abilities [132].

### 9.4. Creatine Supplementation and Cognitive Function in the Heat

Although the role of creatine for reducing muscular fatigue development is well documented during short-duration, high-intensity activities, its role in mitigating central fatigue during exertional-heat stress has received considerably less attention [148]. Hadjicharalambous and colleagues [149] determined the association between brain 5-HT and DA responses and perception of effort during prolonged exercise in the heat following creatine supplementation (20 g·day^−1^ for 7 days; Table 2). The authors alluded to the potential for creatine administration to positively influence key modulators of brain 5-HT and DA function and reduce various thermoregulatory measures [149]. Although this was attributed to a reduced effort perception during exercise in the heat, performance was only improved in “responders” to creatine supplementation [149]. Therefore, this should be considered when assessing the influence of creatine supplementation on individual responses to exertional-heat stress, particularly when focusing on modulators of various brain neurotransmitters. 

**Table 2:** Summary of studies investigating the effect of caffeine, nitrates, tyrosine, and creatine on acute responses to exertional-heat exposure.

Study	Participant Characteristics	Supplementation Protocol	Performance Testing	Outcome
*Caffeine*				
Cohen et al. [95]	Distance runners (*m* = 5, *f* = 2; age, 33 ± 9 years)	Randomly assigned 0, 5, or 9 mg·kg^−1^ of caffeine	3 × 21 km running race (26 °C)	- No significant different in race times between supplementation groups
Ferreira et al. [94]	Highly trained cyclists (*n* = 8)	Randomly assigned 0, 5, or 9 mg·kg^−1^ of caffeine	3 × 45 km cycling race (30 °C; RH, 75%)	- No significant different in race times between supplementation groups
Cheuvront et al. [87]	Healthy males (*n* = 10; age, 23 years)	9 mg·kg^−1^ of caffeine or PLA	Cycling at 50% VO_2peak_ preceding a 15 min TT (40 °C; RH, 20–30%)	- No differences in physiological, perceptual, or performance measures between supplementation groups
Hanson et al. [96]	Endurance runners (*m* = 6, *f* = 4; age, 26 ± 9 years)	Randomly assigned 0, 3, or 6 mg·kg^−1^ of caffeine	3 × 10 km runs (30 °C; RH, 50%)	- No difference in 10 km run time between supplementation groups ↑ Rise in T_c_ high (+0.26 °C) vs. moderate (+0.20 °C) vs. no caffeine (+0.19 °C)
Roelands et al. [97]	Trained male cyclists (*n* = 8; age, 23 ± 5 years)	Randomly assigned 0 or 6 mg·kg^−1^ of caffeine drink	60 min cycle at 55% W_max_, proceeded by a TT (30 °C; RH, 50–60%)	- No difference in performance between supplementation groups ↑ T_c_ during exercise in caffeine supplementation vs. PLA
Ping et al. [99]	Trained male runners (*n* = 9; age, 25 ± 7 years)	Randomly assigned 0 or 5 mg·kg^−1^ of caffeine drink	60 min run at 70% VO_2max_ (31 °C; RH, 70%)	↑ TTE with caffeine (110 ± 29.6 min) vs. PLA (83.6 ± 21.4 min) - No significant differences in physiological or perceptual measures between conditions
***Nitrate***				
Kent at al. [114,115]	Endurance-trained male cyclists (*n* = 12; age, 27 ± 6 years)	8.7 mmol·L^−1^ of BJ or a NO_3_^−^ depleted PLA for 3 days prior	2 × 60 min cycles at 60% VO_2peak_ (33 °C; RH, 49%)	- No significant physiological or performance differences between conditions
McQuillan et al. [116]	Well-trained male cyclists (*n* = 8; age, 25 ± 8 years)	8.0 mmol·L^−1^ of BJ or a NO_3_^−^ depleted PLA for 3 days prior	4 km cycling TT (35 °C; RH, 60%)	- No significant difference in power output between BJ (337 ± 50 W) vs. PLA (336 ± 45 W)
Kuennen et al. [113]	Recreationally active men (*n* = 9; age, 24 ± 1 years)	8.4 mmol·L^−1^ of BJ or a NO_3_^−^ depleted PLA for 6 days prior	3 × 45 min battle marches (41 °C; RH, 20%)	↓ Oxygen cost of exercise in BJ vs. PLA condition ↓ IL-6 and TNF- α in BJ vs. PLA condition - No difference in physiological markers between conditions
Amano et al. [117]	Healthy participants (*m* = 5, *f* = 3; age, 24 ± 4 years)	8.0 mmol·L^−1^ of BJ or a NO_3_^−^ depleted PLA for 3 days prior	30 min cycle at 55% VO_2max_ (30 °C; RH, 50%)	↓ MAP following BJ (103 ± 6 mmHg) vs. PLA (112 ± 6 mmHg) - No significant differences between other physiological or thermoregulatory measures
Fowler et al. [101]	Healthy males (*n* = 11)	9.2 mmol·L^−1^ of BJ or a NO_3_^−^ depleted PLA for 5 days prior	Cycling TTE test (35 °C; RH, 28%)	- No significant differences in physiological or performance measures between conditions
***Tyrosine***				
Tumilty et al. [121]	Healthy males (*n* = 8; age, 32 ± 11 years)	500 mL of sugar-free drink (PLA) or same drink with 150 mg^−1^.kg^−1^ of tyrosine	Cycling to exhaustion at 68% VO_2peak_ (30 °C; RH, 60%)	↑ Exercise capacity with tyrosine vs. PLA (~15%) - No significant differences in physiological or perceptual measures between conditions
Coull et al. [127]	Healthy males (*n* = 8; age, 21 ± 1 years)	High (2 × 150 mg^−1^·kg^−1^), low (2 × 75 mg^−1^·kg^−1^) or no tyrosine	Military-based load carriage protocol (40 °C; RH, 30%)	- No significant differences in physiological or performance measures between conditions
Watson et al. [129]	Healthy males (*n* = 8; age, 23 ± 3 years)	2 × 250 mL of sugar-free drink (PLA) or same drink with 150 mg^−1^·kg^−1^ of tyrosine	1 hr of cycling to exhaustion at 70% VO_2peak_ (30 °C; RH, 50%)	- No significant differences in physiological or performance measures between conditions
***Creatine***				
Mendel et al. [143]	Healthy participants (*m* = 15, *f* = 1; age, 26 ± 2 years)	20 g·day^−1^ of creatine for 5 days or PLA	40 min of cycling to 55% VO_2max_ (36 °C; RH, 29%)	- No negative effect on thermoregulatory measures with creatine ingestion
Kilduff et al. [144]	Endurance-trained males (*n* = 21; age, 27 ± 5 years)	20 g·day^−1^ of creatine for 7 days or PLA	2 × exercise tests to exhaustion at 63% VO_2max_ (30 °C; RH, 70%)	↑ Intracellular water volume and sweat rate in creatine group ↓ Heart rate and Tc in creatine group
Volek et al. [142]	Healthy males (*n* = 20; age, 23 ± 1 years)	20 g·kg^−1^ of creatine for 7 days or PLA	30 min cycling at 60–70% VO_2peak_ followed by 3 x 10 s sprints (37 °C; RH, 80%)	↑ body mass in creatine group (+0.75 kg) - No significant differences in physiological measures between groups
Wright et al. [146]	Physically active males (*n* = 10; age, 26 ± 5 years)	4 × 5 g·day^−1^ of creatine for 6 days or PLA	6 × 10 s sprints on cycle ergometer (35 °C; RH, 60%)	↑ body mass with creatine (+1.30 ± 0.63 kg) vs. PLA (+0.11 ± 0.52 kg) ↑ Power output with creatine vs. PLA - No significant differences in physiological measures between groups
Weiss & Powers [147]	Aerobically trained males (*n* = 24; age, 23 ± 3 years)	5 g·day^−1^ of creatine for 5 days or PLA	60 min cycling at 70% of HR_max_ (37 °C)	↑ Total body water in creatine group - No significant differences in thermoregulatory measures between groups
Watson et al. [145]	Active males (*n* = 12; age, 22 ± 1 years)	26.1 g·day^−1^ of creatine for 7 days or PLA	80 min exertional-heat stress test (34 °C; RH, 41%)	- No impairment in hydration status or thermoregulatory function with creatine ingestion
Hadjicharalambous et al. [149]	Endurance-trained males (*n* = 21; age, 27 ± 5 years)	20 g·day^−1^ of creatine for 7 days or PLA	Exercise tests to exhaustion at 63% VO_2max_ (30 °C; RH, 70%)	↑ Exercise capacity with creatine vs. PLA (responders only) ↑ Modulators of brain 5-HT and DA function with creatine supplementation

RH, Relative humidity; PLA, Placebo; T_C_, Core temperature; W_max_, Maximum power; TT, Time trial; TTE, Time-to-exhaustion; BJ, Beetroot juice; NO_3_^−^, Nitrate; VO_2peak_, Peak oxygen uptake; W, Watts; IL-6, Interleukin 6; TNF-α, Tumour necrosis factor alpha; mmHg, Millimetres of mercury; VO_2max_, Maximal oxygen uptake; HR_max_, Maximal heart rate; 5-HT, serotonin; DA, dopamine.

## 10. Limitations

Despite the practical relevance of sport nutrition topics discussed, there are some limitations that may influence the scientific consistency of this review. Due to the nature of narrative reviews, which have potential selection bias as a result of a lack of structured criteria for selecting and analysing studies, there is potential risk of selection bias in the studies chosen for discussion. However, a predetermined search strategy, with a specified inclusion and exclusion criteria was applied to reduce the risk of study selection bias. In line with this, although the quality of the studies was not assessed using a specific risk of bias tool, the methodologies, findings, and application of specific nutritional strategies and ergogenic aids in included studies were critically discussed within this review. Lastly, differing supplementation doses and timings, training status of participants, and the sensitivity and specificity of performance measures adopted across studies present difficulties proposing accurate guidelines for the prescription of nutritional strategies and ergogenic aids for exertional-heat stress. As such, the authors note that recommendations made within this review for prescribing tailored nutritional strategies and ergogenic aids to specific population groups should be interpreted with caution due to the aforementioned limitations. Despite this, careful consideration was taken in the discussion and suggested use of various nutritional strategies and ergogenic aids to optimise performance and health in different exercise settings in the heat, as the authors aimed to present this review in the most objective manner possible.

## 11. Conclusions

**Figure 1:**
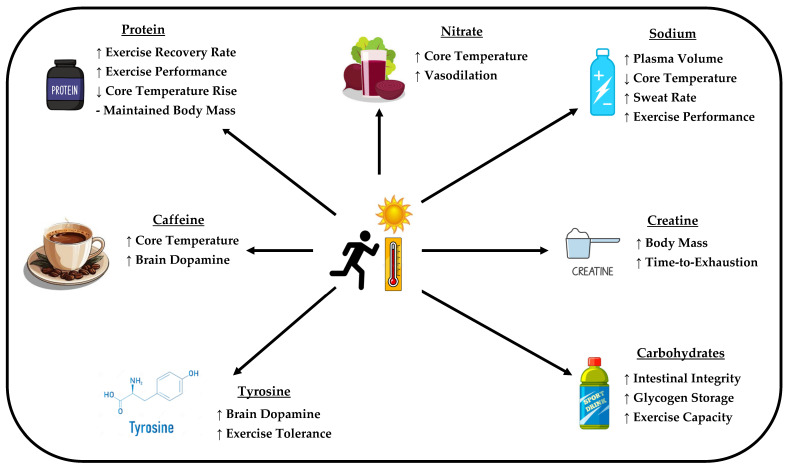
Schematic representing the effect of different ergogenic aids on acute responses and chronic adaptations to exertional-heat exposure.

Research surrounding nutritional strategies and ergogenic aids for enhancing physiological, cognitive, and recovery outcomes for exercise has received considerable attention. To the best of the authors’ knowledge, this was the first review to summarise the influence of different nutritional practices and ergogenic aids on responses to acute and chronic exertional-heat exposure. As exercise in the heat alters the efficacy of numerous dietary supplements, the results of certain ergogenic aids in thermoneutral conditions do not always transfer to conditions of high ambient temperatures. Equivocal findings across studies assessing the efficacy of ergogenic aids on acute and chronic exertional-heat exposure are likely dependent on exercise testing modality, duration, and intensity, outcome measures in relation to the ergogenic aid’s proposed mechanism of action, and sex-specific responses to supplementation. Empirical evidence exists to propose sodium ingestion for increasing P_V_ and improving exercise performance in the heat. Although the optimal sodium dose has not yet been established, acute doses of 20–40 mg·kg^−1^ in the 1–2 h prior to acute exertional-heat exposure have demonstrated beneficial effects. Furthermore, tailored exogenous carbohydrate strategies can enhance exercise capacity in the heat whilst maintaining intestinal wall integrity. The suggested carbohydrate dose is ~30–90 g·h^−1^ during prolonged exercise in the heat; however, males may benefit from carbohydrate ingestion that is closer to the upper limit of the suggested dose due to their higher relative carbohydrate utilisation rates during exercise compared to females. The additive use of protein to carbohydrate intake may further augment P_V_ during HA/HAs, in addition to accelerating recovery following exertional-heat exposure. Although the addition of protein may not be necessary to elicit performance improvements when the delivery of carbohydrates is optimal (≥60·g·h^−1^), it may still be beneficial for skeletal muscle recovery following exertional-heat exposure, especially in females experiencing reductions in muscle protein synthesis due to ovarian hormone fluctuations. Heat-mediated decrements in cognitive function may be reduced following caffeine (~6 mg·kg^−1^, 1–2 h prior to exercise) and creatine consumption (20 g·day^−1^ for ~7 days or 5 g daily), with the influence on performance likely related to the exercise modality, duration, and intensity adopted in certain studies, in addition to interindividual differences in caffeine metabolism. Furthermore, caffeine intake may increase T_c_, whilst creatine does not appear to exacerbate thermoregulatory strain. Lastly, the proposed mechanism through which NO_3_^−^ and tyrosine may improve exercise performance in the heat requires further study, with the majority of positive findings only being exhibited in studies conducting testing in thermoneutral conditions (Figure 1). However, if individuals chose to utilise these ergogenic aids, 150 mg·kg^−1^ of tyrosine appears sufficient to maximise plasma tyrosine levels, with ~8 mmol·L^−1^ of nitrates being recommended to observe any potential benefits. Future research should (1) address dose–response relationships of ergogenic aids for various physiological and cognitive performance outcomes during and following exertional-heat exposure, (2) identify sex- and exercise modality/sport-specific responses following supplementation for exercise performance in the heat, and (3) utilise appropriate performance measures in relation to the supplement’s proposed mechanisms of action. Collectively, this review summarises evidence that can help to prescribe tailored nutritional strategies for populations frequently exposed to exertional-heat stress, with the overall goal of optimising performance and health outcomes.
